# Myoepithelioma-like hyalinizing epithelioid tumor of the foot with OGT-FOX03 fusion gene: Imaging findings, surgical implications, and pathological correlates

**DOI:** 10.1016/j.radcr.2022.12.014

**Published:** 2022-12-25

**Authors:** Kimberly Boldig, Matthew Montanarella, Weibo Fu, Jennifer M. So, Jacqueline C. Lucke, Kristin Taylor, Jason A. Piraino, Abhinav Rohatgi

**Affiliations:** a Department of Medicine, University of Florida College of Medicine Jacksonville, 655 W 8th St, Jacksonville, FL 32209, USA; b Department of Radiology, University of Florida College of Medicine Jacksonville, Jacksonville, FL 32209, USA; c Division of Foot and Ankle Surgery, Department of Orthopedics and Rehabilitation, University of Florida College of Medicine Jacksonville, Jacksonville, FL 32209, USA; d Department of Hematology and Oncology, University of Florida College of Medicine Jacksonville, Jacksonville, FL 32209, USA

**Keywords:** Myoepithelioma-like hyalinizing epithelioid tumor, OGT-FOX03, MRI, Myoepithelioma

## Abstract

Myoepithelioma-like hyalinizing epithelioid tumors are rare neoplasms that share morphological characteristics of myoepitheliomas but lack traditional immunophenotypic findings. Though little is known about these tumors at present, a handful of recent studies have confirmed that they harbor a novel fusion gene known as “OGT-FOXO.” Though closely resembling myoeptheliomas, Myoepithelioma-like hyalinizing epithelioid tumors are considered a distinct tumor entity, and few studies have explored their clinical characteristics or their potential for malignancy. Furthermore, literature describing imaging findings of these tumors is virtually non-existent. Understanding the radiological and pathological differences between Myoepithelioma-like hyalinizing epithelioid tumors and myoepitheliomas is helpful in developing a comprehensive differential for soft tissue neoplasms of the foot. We describe a case of MHET of the foot and correlate MRI findings with pathology in addition to describing surgical technique and implications to care.

## Introduction

Myoepithelial tumors are uncommon, soft-tissue neoplasms which are commonly found in the soft tissue of the extremities and limb girdles. They affect men and women equally, with mean age of incidence occurring at 40 years [1]. These tumors most commonly present as a painful mass in the extremities in subcutaneous or deep soft tissue [2]. Due to their rarity, criteria for malignancy have not been clearly established, and staging for malignant myoepitheliomas is often determined on a case-by-case basis [3,4]. Histologically, both benign and malignant myoepithelial neoplasms are usually well-circumscribed, unencapsulated, and have infiltrative margins [1,4]. These neoplasms are commonly composed of epithelioid, or spindle cells arranged in different architectures [4,5]. Genetically, more than half of myoepithelial tumors harbor the ESWR1 gene [5].

Diagnosis of most tumors requires tissue biopsy, assessment of morphology, immunohistochemistry (IHC), and genetic analysis once a mass is identified via physical exam and/or imaging [5]. More recently, a new tumor known as a myoepithelioma-like hyalinizing epithelioid tumor (MHET) has been identified in the literature; its name alludes to its phenotypic resemblance to a myoepithelioma despite not meeting criteria on IHC for diagnosis. Like myoepitheliomas, MHETs have an epithelioid phenotype and sclerotic background [6] However, their lineage is separate from myoepitheliomas and remains to be determined. Furthermore, MHETs harbor a previously unidentified fusion gene, most recently described in a 2020 study as “OGT-Forkhead Box.” or OGT-FOXO fusion gene [5,6]. As a new tumor lineage, very little is known about imaging or clinical features of MHETs. The goal of this case report is to describe a case of MHET of the foot and correlate imaging findings with pathology.

The Forkhead box O-class subfamily includes transcription factors FOXO1, FOXO3, FOXO4, and FOXO6. FOXO proteins are tumor suppressor genes that regulate cellular differentiation, apoptosis, and cell proliferation. O-GlcNAc transferase (OGT) gene is an enzyme involved in protein glycosylation, regulating gene transcription, protein stabilization, and degradation. It is believed that the OGT-FOXO fusion causes glycosylation of FOXO, increased OGT activity, and possibly tumor growth through this mechanism [7].

## Case presentation

**Fig. 1:**
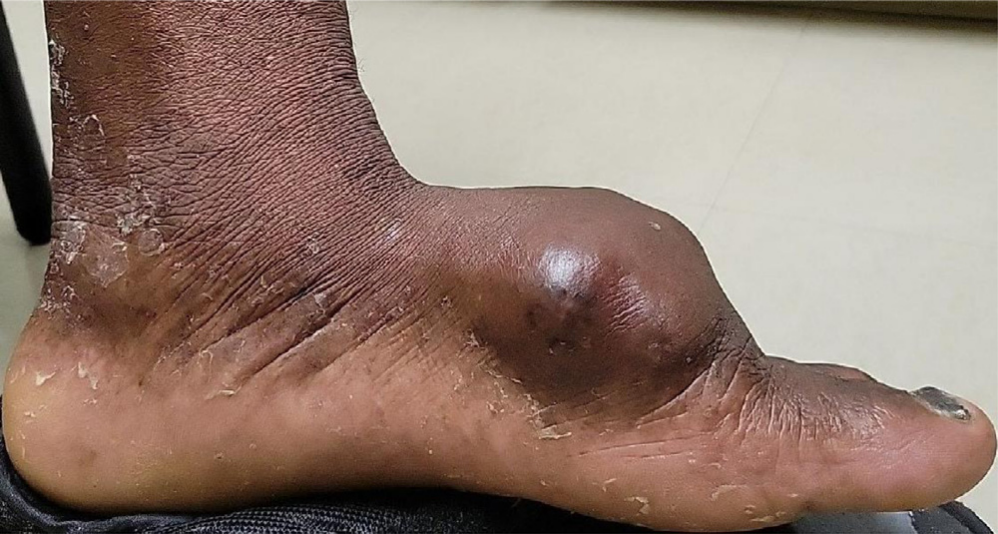
Photograph demonstrating a large mass over the dorsum of the left foot.

A 58-year-old male initially presented to his podiatry clinic with foot pain 9 months after noticing a mass on the dorsum of his left foot (Fig. 1).

**Fig. 2:**
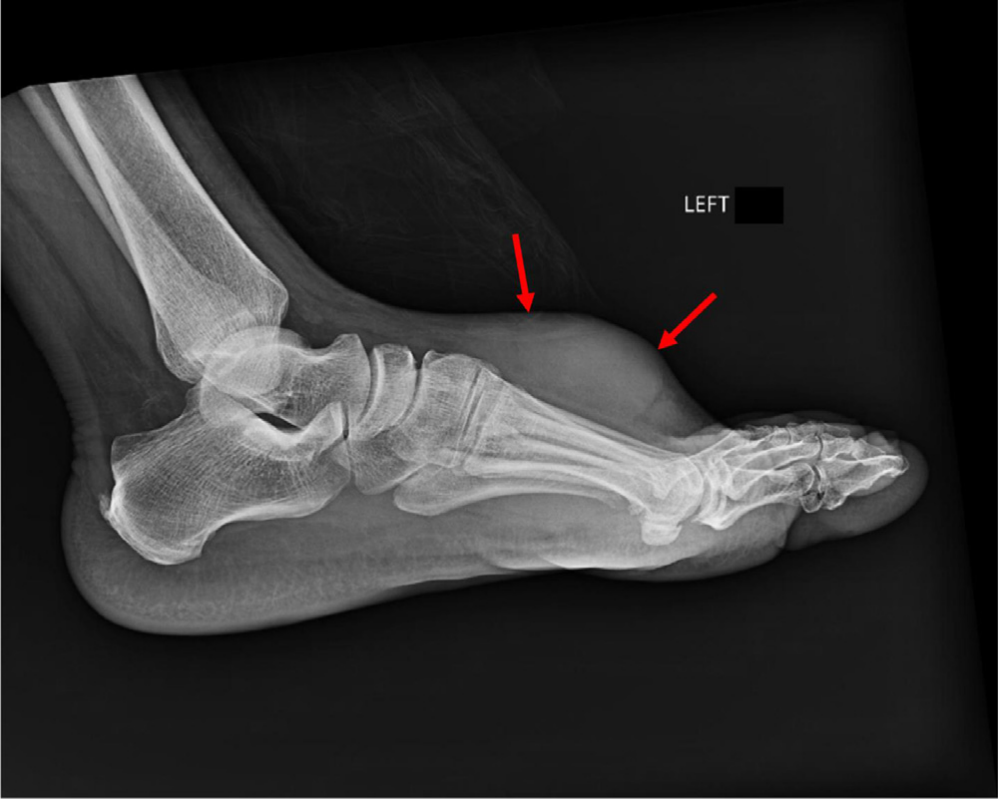
Radiograph of the left foot in a lateral projection demonstrating a large soft tissue mass over the dorsum of the foot (arrows). There is no evidence of bony erosion or fractures.

The patient reported that the mass quickly grew over the few months prior and was now associated with pain. Associated symptoms included numbness, tinxgling, and difficulty wearing shoes. The severity of pain worsened over time, and the patient eventually required a cane to ambulate. During his visit, an X-ray was performed, which demonstrated a soft tissue mass along the dorsal aspect of the medial mid foot (Fig. 2).

**Fig. 3:**
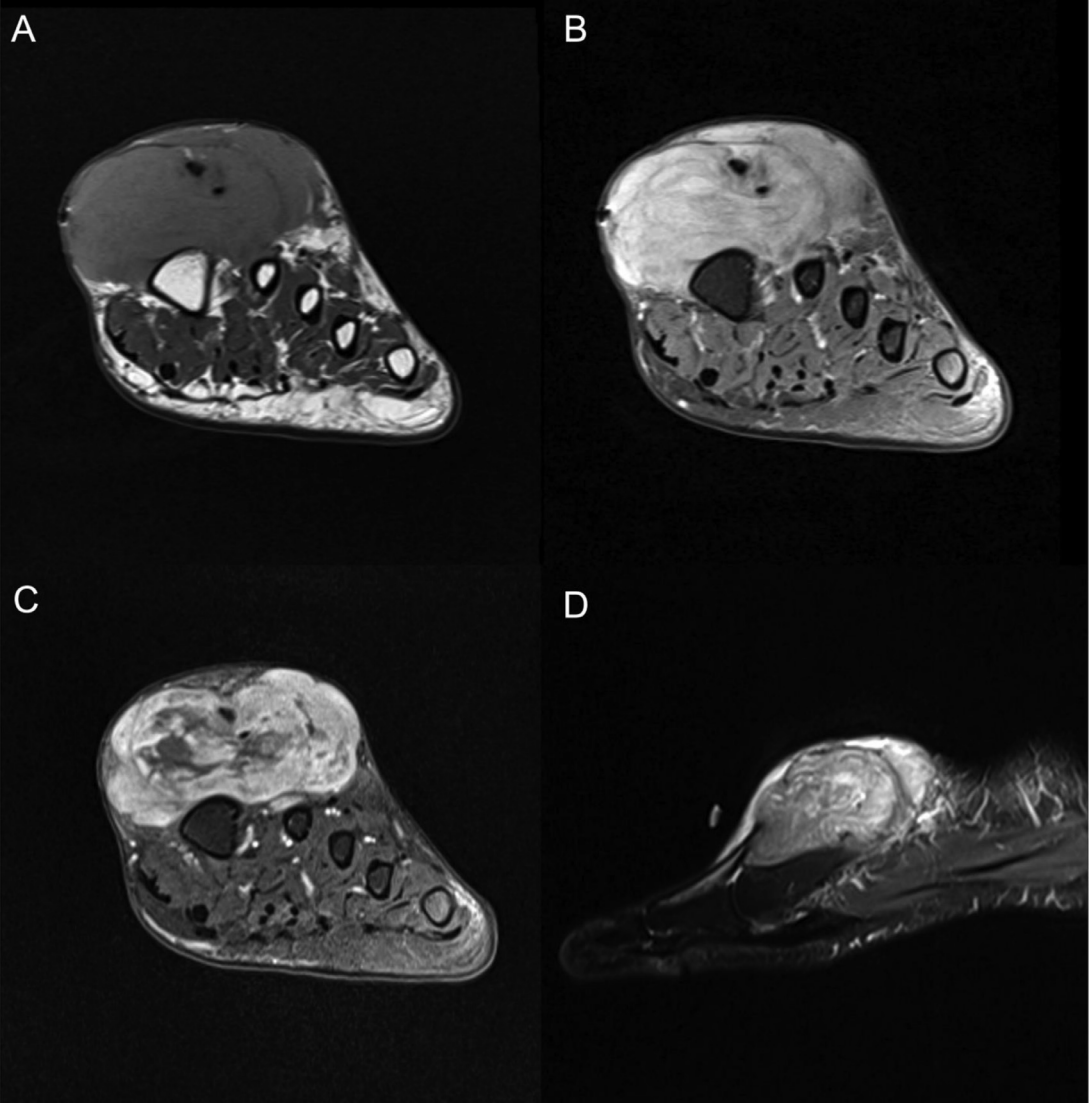
(A) Short axis T1 sequence demonstrating an intermediate T1 signal mass with encasement of the extensor hallucis longus/brevis tendons. (B) Short axis proton density fat saturation (PDFS) sequence demonstrating uniformly increased signal without internal fat. (C) Short axis T1 fat saturated contrast enhanced sequence demonstrating heterogeneous enhancement with multifocal regions of non-enhancement corresponding to the cystic component. (D) Sagittal short tau inversion recovery (STIR) sequence confirming the internal cystic areas within the lesion causing mass effect on the dorsal tendons.

An MRI was ordered for further characterization. However, the patient had not followed up and subsequently presented to the ED 6 months after the initial presentation. At that time, the tumor had been present for about 1.5 years with significant interval growth, decrease in digital range of motion (ROM), interfering with the patient's activities of daily living. The MRI demonstrated an enhancing mass measuring 2.6 × 6.0 × 6.9 cm along the dorsum of the midfoot surrounding the extensor hallucis longus (EHL) and extensor hallucis brevis (EDB) tendons (Fig. 3).

**Fig. 4:**
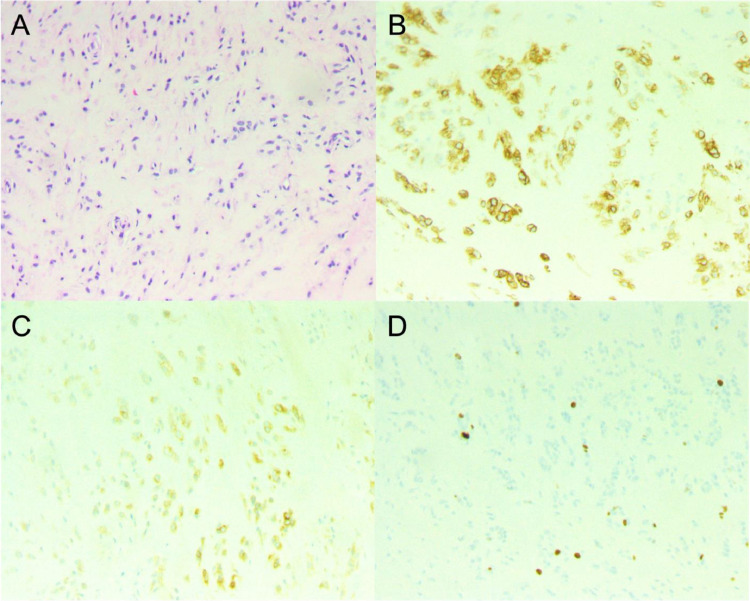
Histology and immunohistochemical evaluation of the tumor. (A) Bland spindle /epithelioid cells arranged in a chondromyxoid background with scattered collagen (H&E stain, ×10). (B) Tumor cells are diffusely positive for CD34 (×10). (C) Tumor cells are positive for EMA (x10). (D) Tumor cells show low Ki-67 proliferation index.

The differential diagnosis included left foot desmoid tumor, giant cell tumor of tendon sheath, rhabdomyosarcoma, synovial sarcoma among others. The patient was promptly referred to hematology and oncology and scheduled for a biopsy of the mass, which demonstrated findings consistent with a myoepithelioma-like hyalinizing epithelioid tumor. Histologic examination showed an epithelioid neoplasm arranged in clusters and cords in a chondromyxoid background with scattered collagen deposition. There was no significant cytologic atypia, mitotic figures, or necrosis (Fig. 4).

Molecular profiling identified OGT-FOXO3 fusion. Cells were found positive for CD34 and EMA. They were negative for S100, SOX10, SMA, desmin, MSA, CD31, beta-catenin, AE1/AE3, and Ki-67 proliferation index is low. The cells were also negative for PANCK, MUC4, TLE1, GFAP, ALK-1. The tumor lacked the typical immunohistochemical features of myoepithelial lineage.

**Fig. 5:**
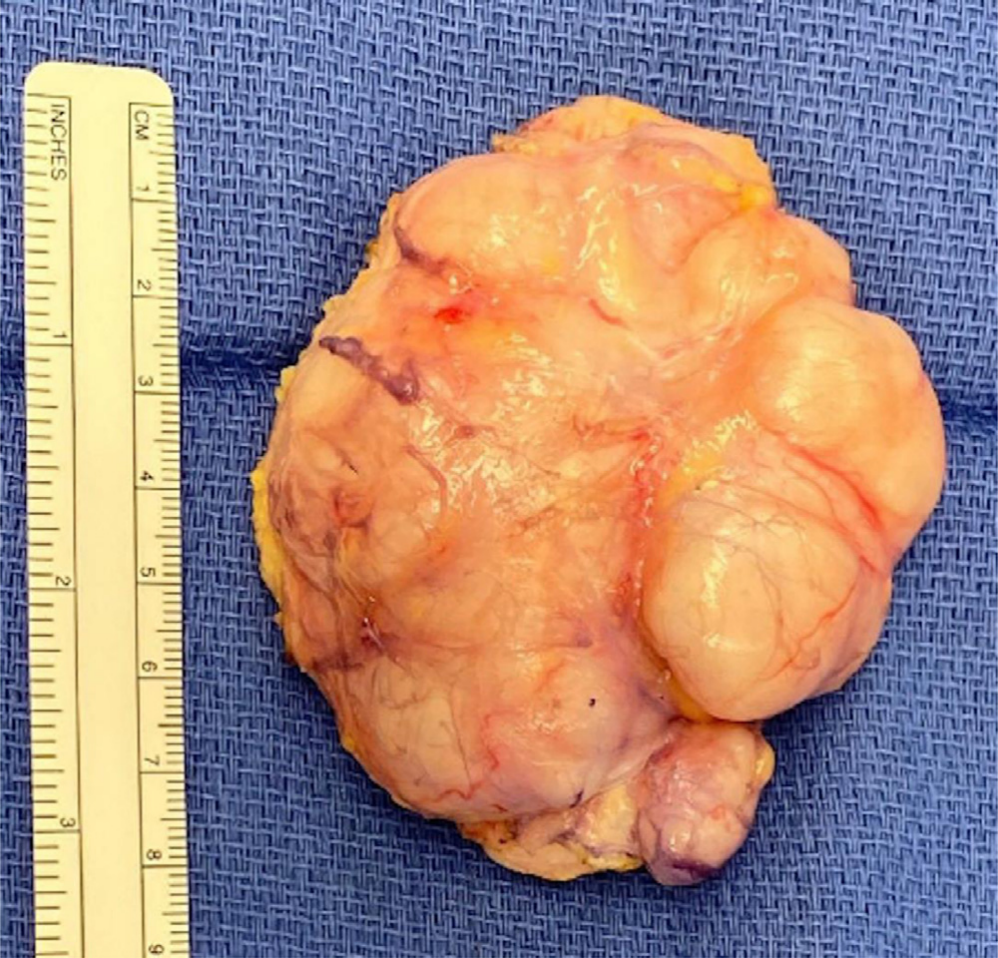
Gross specimen of the myoepithelioma-like hyalinizing epithelioid tumor following surgical resection.

Fluorescence in Situ Hybridization (FISH) studies were negative for EWSR1 and NR4A3 which excluded the diagnosis of extraskeletal myxoid chondrosarcoma. Although the tumor appeared benign on histologic examination, it had an unknown malignant potential. The patient was presented options for amputation versus mass resection. The patient elected to undergo operative excision of the mass (Fig. 5).

### Surgical technique

Surgical intervention was staged in 2 parts and consisted of core decompression biopsy by the Division of Foot and Ankle Surgery and definitive excision by the Orthopedic Oncology Service.

### Needle biopsy

Percutaneous skin incision was made over the largest lobe of the tumor at the dorsal medial foot. Blunt dissection through subcutaneous tissue and sharp incision through the tumor capsule was performed. Superficial and deep biopsies of the tumor were obtained and sent in formalin for pathologic analysis.

### Definitive excision

A 9-cm longitudinal skin incision was made over the mid-dorsal aspect of the mass. The mass's capsule was incised in line with skin incision and bluntly released from surrounding soft tissues. The dorsal pedal neurovascular bundle was identified inferior and lateral to the mass, and subsequently protected. A cobb elevator was used to resect the mass which abutted underlying bone. The EHL and EDB tendons had been fully encapsulated by the mass and were sacrificed during mass excision. The mass was removed en toto and sent to pathology for final analysis. The mass measured approximately 6.0 × 7.0 × 3.0 cm (Fig. 5). This was consistent with the MRI measurements of 6.0 × 6.9 × 2.6 cm. Remaining excess skin was removed with ellipse incision, and the skin was closed primarily. The patient was splinted for immobilization.

## Discussion

### Imaging features

This MHET diagnosis has only been described 4 times previously in the literature and descriptive imaging findings were never reported [5,^6^,8]. MRI features of a soft tissue myoepitheliomas have been described in the literature. Therefore, we used these for comparison because they harbor a traditional immunophenotype. In a case of myoepithelioid tumors of the lateral abdominal wall, MRI demonstrated heterogeneous intermediate T1 signal and high T2 signal [2]. Most of the cystic areas in the lesion were T2 bright and demonstrated well circumscribed borders [2]. In a retrospective study of 5 parotid myoepitheliomas, all exhibited homogeneous isointense signal on T1-weighted imaging [9]. On T2-weighted imaging with fat suppression all 5 tumors exhibited homogeneous intermediate to high signal intensity [9]. In a case of malignant scrotal myoepithelioma, T2-weighted MRI demonstrated a heterogeneous area of intermediate signal containing low signal reticular structures on T2-weighted imaging [10]. On T1-weighted imaging parts of the tumor had markedly high signal compared to muscle, but no fatty components were visible on fat-saturated sequences [10]. Additional documented cases of soft tissue MHETs were consistent with well-defined masses with low to intermediate signal and had internal high signal areas, likely representing internal hemorrhagic areas [11], [12], [13]. With no definitive imaging characteristics to differentiate benign and malignant myoepitheliomas, it was concluded that tissue biopsy was essential to diagnosis [10].

Our case of MHET demonstrated intermediate T1 signal and high T2 signal with cystic components, imaging findings that were consistent with those described above, which posed challenge in developing a differential diagnosis. With no distinctive imaging findings to differentiate MHETs from myoepitheliomas, tissue biopsy was necessary for diagnosis. Biopsy results of our patient revealed no cytologic atypia, mitotic figures, or necrosis, indicating benign tumor characteristics. Identifying the novel OGT-FOX03 fusion gene and absence of typical pathognomonic immunophenotypic supporting features of myoepitheliomas allowed for diagnostic confidence.

### Pathology

Pathology of previously reported myoepithelioma-like hyalinizing epithelioid tumors describe well demarcated tumors with hyalinizing to myxoid stroma and epithelial tumor cells. A matrix component consisted of eosinophilic collagen. Epithelioid tumor cells with uniform, rounded nuclei were arranged in cords or nests [5]. Mitotic activity was very low. The cellular processes often encircled hyalinized stromal material, giving a vacuolated lumen-like appearance. Spindle cells with fibrous stroma were also seen. The 2 tumors described also had rich vasculature [5]. They were both found to be focally positive for EMA and diffusely positive for CD34. Immunohistochemical staining for S100, GFAP, p63, SMA, GLUT1, claudin-1, CD31, and ERG were negative. Both cases also had OGT-FOXO3 gene fusion [5]. An additional case report described epithelioid and spindled neoplastic cells with eosinophilic cytoplasm. The nuclei had mild atypia and the tumor lacked mitotic activity and necrosis. In this case the tumor was positive for EMA, pan-CK, CD34, ERG, and FLI1. It was negative for CD31, S100, SOX 10, desmin, MUC4. The OGT-FOXO4 fusion gene was identified [6]. The fourth case described in literature again describes epithelioid and spindle cells with hyalinizing or myxoid stroma. Nuclear atypia and mitotic figures were minimal. The cells were positive for CD34 and negative for AE1/AE3, S100, smooth muscle actin, GLUT1, and claudin-1. An OGT-FOXO1 fusion was found [8]. These 4 cases reiterated the pathologic and immunohistochemical findings in our case report.

The pathology relating to these myoepithelioma-like hyalinizing epithelioid tumors demonstrates a benign nature of the tumor. In a study of 401 soft tissue tumors, Kaposi's sarcoma and synovial sarcoma were the most common malignancies of the foot [14]. This study also study concluded that there may be a biological spectrum of benign and malignant tumors of the foot with significant overlap in presentation clinically, potentially leading to a delay is diagnosis of up to 21 months [14]. A better understanding of the malignant potential of myoepithelial-like hyalinizing epithelioid tumors important for clinical practice.

### Surgical implications

Surgical intervention was staged with a needle biopsy followed by definitive excision. The initial surgical biopsy helped determine the treatment plan and prognosis for limb preservation. At most recent follow up, the patient was overall doing well with some reported paresthesia to dorsal foot and difficulty with active ROM of digits 1-3 secondary to EHL/EDL tendon sacrifice. Due to the rarity of this tumor, the most immediate concern is local recurrence. The consensus recommendation between orthopedic oncology, medical oncology, and pathology was for a repeat MRI and clinical exam in 6 months.

## Conclusion

MHETs are an extremely rare distinct tumor entity from myoepithelial tumors which harbor a novel OGT-FOX03 fusion gene. There are no distinctive MRI findings that can differentiate them from myoepithomas in the soft tissue. Tissue biopsy is required for definitive diagnosis of these tumors. Future studies with patient follow up of documented cases are required to better elucidate their malignant potential and establish standardized treatment guidelines.

## Author contribution

All authors contributed to project conception, manuscript preparation, and manuscript editing.

## Patient consent

A written consent was obtained from the patient for publication of this case and any accompanying images.
